# Enzymatic Synthesis of a Novel Antioxidant Octacosanol Lipoate and Its Antioxidant Potency in Sunflower Oil

**DOI:** 10.1021/acs.jafc.4c07240

**Published:** 2024-09-18

**Authors:** Wen-Sen He, Liying Zhao, Jiawei Sui, Xian Li, Shouhe Huang, Huafang Ding, Hanyue Zhu, Zhen-Yu Chen

**Affiliations:** † School of Food and Biological Engineering, Jiangsu University, Zhenjiang, Jiangsu 212013, China; ‡ School of Life Sciences, The Chinese University of Hong Kong, Shatin, Hong Kong 999077, China; § School of Food Science and Engineering/Guangdong Provincial Key Laboratory of Intelligent Food Manufacturing, Foshan University, Foshan, Guangdong 528000, China

**Keywords:** lipoic acid, octacosanol, Candida sp. 99−125 lipase, antioxidant, ^1^H nuclear magnetic resonance

## Abstract

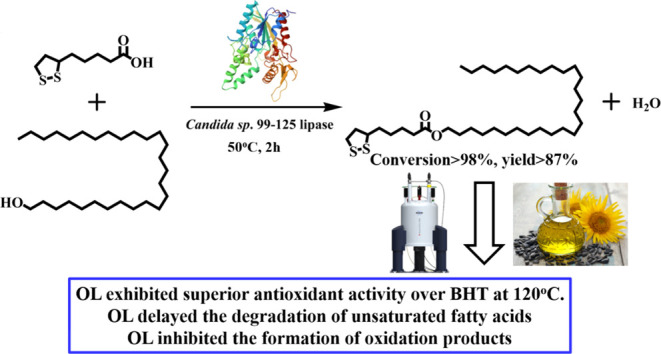

α-Lipoic acid possesses remarkable antioxidant activity; however, its poor lipid solubility greatly restricts its practical utilization. The present study was the first (i) to synthesize a novel lipophilic antioxidant of octacosanol lipoate and (ii) to assess its antioxidant potency in sunflower oil by hydrogen nuclear magnetic resonance (^1^H NMR) spectroscopy. In brief, octacosanol lipoate was successfully synthesized using octacosanol and lipoic acid as substrates and *Candida sp*. 99–125 lipase as a catalyst. The conversion of octacosanol lipoate could reach as high as 98.1% within merely 2 h, with an overall yield of 87.9%. The hydrophobicity of lipoic acid was significantly enhanced upon esterification with octacosanol. Interestingly, both traditional methods and ^1^H NMR analysis consistently indicated that octacosanol lipoate exhibited superior antioxidant activity compared with butyl hydroxytoluene at high temperatures. It was concluded that octacosanol lipoate has the potential to be developed into a safe and efficient natural antioxidant which can be utilized not only in daily cooking oils but also in frying oils.

## Introduction

Oxidation remains a primary factor contributing to the deterioration of oil and fat. The oxidation process not only results in unpleasant flavors and diminished nutritional value but also generates potentially harmful substances like peroxides, aldehydes, and ketones.^1−3^ To address this issue, nitrogen-filled preservation and vacuum packaging technologies are gaining popularity as innovative methods to retard the oxidation of fats and oils. Although these methods avoid the use of external additives, they are significantly more expensive. Moreover, their protective efficacy is compromised once the packaging is breached. Currently, antioxidants are commonly employed. However, widely used antioxidants are predominantly chemically synthesized, such as butyl hydroxytoluene (BHT) and butyl hydroxyanisole (BHA). There are always concerns about their safety, particularly regarding their potential subchronic toxicity and possibly carcinogenesis at higher concentrations tested in rodents.^4−6^ Therefore, the development of natural antioxidants with high safety and antioxidant activity is imminent.

α-Lipoic acid is a disulfide compound that is naturally synthesized in humans and found in foods, such as meats, tomatoes, broccoli, carrots, and other plants.^7^ It is known that α-lipoic acid is essential for mitochondrial aerobic metabolism. In addition, numerous studies have demonstrated that α-lipoic acid exhibits a range of other biological activities, including antioxidation, anti-inflammation,^8^ antidiabetes,^9^ neuroprotection,^10^ amelioration of respiratory system diseases,^11^ cholesterol reduction,^7^ and antiobesity.^12^ It is extensively utilized as both a medicinal compound and a dietary supplement.^13^ Notably, α-lipoic acid exhibits a remarkable antioxidant activity and is commonly known as a “universal antioxidant” due to its high safety profile and no or minimal side effects.^14^ However, α-lipoic acid is susceptible to light and heat,^15^ and its solubility in lipids is poor, thereby limiting its application in oils and fats and hindering its full potential as an antioxidant.^16^

Octacosanol is an aliphatic alcohol that is naturally found in wheat germ, sugar cane wax, and beeswax.^17^ It possesses several significant biological activities, including antifatigue,^18^ lipid reduction,^17^ prevention of cardiovascular diseases,^19^ and anti-inflammation.^20^ Combining α-lipoic acid and octacosanol through chemical bonding not only enhances the lipid solubility and stability of α-lipoic acid but also imparts the physiological activities of both compounds. The synthesis of α-lipoic acid ester can be achieved through a chemical or enzymatic route. While a chemical method offers some advantages such as high efficiency and low cost, they suffer from drawbacks such as complex steps, challenging product separation, and harsh reaction conditions.^21,22^ Due to the instability of α-lipoic acid, a milder synthesis route is required. An enzymatic route, as a novel and environmentally friendly green synthesis technology, presents several advantages, such as mild reaction conditions, high selectivity, and easy product separation.^21^ To the best of our knowledge, no attempt to synthesize octacosanol lipoate has yet been reported.

Conventional methods for assessing oil oxidation, such as the peroxide value, anisidine value, and carbonyl value, are known for their complexity, time-consuming nature, and elevated expenses. Oxidation is a multifaceted and dynamic process, making it challenging for a single metric to accurately capture the extent of oxidation. Nuclear magnetic resonance hydrogen spectrum analysis (^1^H NMR) utilizes the magnetic properties of hydrogen nuclei to detect changes in proton chemical shifts that occur during oxidation.^23^ This allows for precise measurement of fatty acids and both primary and secondary oxidation products, providing a comprehensive understanding of the oxidation status of the oil. With its rapid analysis, efficiency, precision, reproducibility, straightforward pretreatment requirements, and minimal sample volume, ^1^H NMR method can serve as a new alternative for assessing the oxidation of fats and oils.^24^

The present study was therefore conducted (i) to synthesize octacosanol lipoate via a lipase-catalyzed esterification process for the first time and (ii) to evaluate its antioxidant potency in vegetable oil by ^1^H NMR spectroscopy.

## Materials and Methods

### Materials

Lipoic acid and butyl hydroxytoluene (BHT) (>99%) were purchased from Aladdin Co. Ltd. (Shanghai, China). Octacosanol (>95%) was provided by Changsha Nurtritopper Biotechnology Co., Ltd. (Changsha, China). The lipases *Candida sp*. 99–125 (20,000 U/g), *Candida rugosa* (Type VII, 700 U/mg), and Lipozyme RM IM (275 IUN/g) were acquired from Beijing CTA New Century Biotechnology Co. Ltd. (Beijing, China), Sigma Co. Ltd. (Shanghai, China), and Novozymes China Biotechnology Co. Ltd. (Tianjin, China), respectively. Other reagents were of analytical grade and provided by Sinopharma Chemical Reagents Co. Ltd. (Shanghai, China).

### Enzymatic Synthesis of Octacosanol Lipoate

Lipoic acid (0.04–0.10 g), octacosanol (0.08–0.20 g), lipase (0.025–0.175 g), activated 3 Å molecular sieve (0.60 g), and reaction solvent (5 mL) were introduced into a 20 mL brown reaction bottle in sequence. The resulting mixture was transferred to an air bath shaker, which was set to maintain a temperature of 35–60 °C and allowed to react for a duration of 2–6 h. Periodically, 100 μL samples were taken from the reaction mixture for the analysis of thin-layer chromatography (TLC) and high-performance liquid chromatography (HPLC). TLC was used for quick qualitative identification of octacosanol lipoate, while HPLC was applied for the quantification of octacosanol lipoate. To ensure the reliability and reproducibility of the results, all experiments were conducted at least 3 times.

### Measurement of Residual Enzyme Activity

The 0.5 g portion of *Candida sp*. 99–125 lipase was added to a brown reaction bottle containing 5 mL of isooctane, *n*-hexane, toluene, acetone, or *tert*-pentanol, and the mixture was then subjected to a 40 °C air bath shaker for a duration of 24 h. Following this, the bottle was transferred to a 40 °C vacuum dryer for an additional 24 h to evaporate the solvent, resulting in the treated enzyme. The conversion rate of *Candida sp*. 99–125 lipase after treatment with various solvents was determined under the following reaction conditions. Treated *Candida sp*. 99–125 lipase (0.051 g) was added to a reaction system containing 0.051 g of lipoic acid, 0.103 g of octacosanol, 0.61 g of 3 Å molecular sieve, and 5 mL of isooctane. The system was allowed to react for 2 h at a temperature of 40 °C.

### TLC Analysis of Octacosanol Lipoate

The sample periodically withdrawn was diluted with a mixture of *n*-hexane and isopropanol (1:1, v/v) and subsequently analyzed by TLC employing petroleum ether (60–90 °C)/ethyl acetate (9:1, v/v) and iodine vapor as developing solvent and visualizing agent, respectively.

### Silica Gel Column Chromatography of Octacosanol Lipoate

Following the completion of the reaction, the supernatant was collected via centrifugation, and the solvent was subsequently evaporated to obtain the crude product. This crude product was then subjected to separation using a silica gel column (80 cm × 2.5 cm) with petroleum ether (60–90 °C) and ethyl acetate (9:1, v/v) as the eluent. Throughout the process, TLC was employed to monitor the elution of the product, ensuring that only the pure product was collected. The actual yields of the products were measured under the optimal reaction conditions as described previously.^16^

### HPLC Analysis of Octacosanol Lipoate

The product was quantitatively analyzed according to our previous method with a minor modification. The mobile phase consisted of methanol and 2-propanol (6:4, v/v) at a flow rate of 1.0 mL/min. Quantitative calculations were carried out according to our previous method.^25^

### Structural Characterization of Octacosanol Lipoate

The purified product underwent structural characterization through Fourier transform infrared spectroscopy (FT-IR), high-performance liquid chromatography–mass spectrometry (HPLC-MS), and NMR analysis as previously described. In FT-IR analysis, attenuated total reflection was employed with a scanning range of 600–4000 cm^–1^, 32 scans, and a resolution of 4 cm^–1^. The MS was conducted in electrospray positive-ion mode, with a mass scan range of 50–800 amu, and other parameters remained as previously reported.^26^ For NMR analysis, tetramethylsilane and deuterated chloroform were used as an internal standard and solvent, respectively. The frequency was set at 400 MHz for ^1^H spectra and 100 MHz for ^13^C and DEPT-135 spectra.^16^

### Determination of Melting Points and Oil–Water Partition Coefficients of Octacosanol Lipoate

The melting points of lipoic acid, octacosanol, and octacosanol lipoate were determined using a differential scanning calorimeter. The temperature was raised from 20 to 170 °C at a rate of 10 °C/min. The oil–water partition coefficients (log *P* values) of lipoic acid and octacosanol lipoate were predicted using the Chem 3D 20.0.0.41 software.^27^

### Antioxidant Activity of Octacosanol Lipoate

The inhibitory impact of octacosanol lipoate on lipid oxidation, induced by heat, was assessed by modifying our established method.^23^ Specifically, 3.0 g of fresh sunflower oil was supplemented with 200 ppm of butylated hydroxytoluene (BHT), or an equimolar amount of octacosanol lipoate (OL), or no antioxidant added (CT), and then loaded into sealed test tubes (10 × 1.5 cm^2^). The oil samples were then subjected to heat treatment at 60 °C for 7 and 14 days and at 120 °C for 12 and 24 h, respectively. These tubes were opened for 3 min every 12 h to ensure sufficient oxygen entry. A 0.05 g aliquot of the oil sample was dissolved in 0.6 mL of deuterated chloroform and subjected to ^1^H NMR analysis on an AVANCE II spectrometer, operating at a frequency of 400 MHz. The efficacy of OL in retarding lipid oxidation was evaluated by monitoring the formation of oxidation products and the degradation of unsaturated fatty acids.

Concurrently, the peroxide value (POV) of the oil samples was determined using a method previously described.^28−30^ The anisidine value (*p*-AnV), measured in accordance with the Chinese national standards (GB/T 24304–2009), provided additional insights into the oxidation status. All samples were replicated 3 times in parallel and stored at −20 °C prior to analysis. The total oxidation (TOTOX) value, a comprehensive indicator of oxidation level, was calculated using the formula: TOTOX = 2 × POV + *p*-AnV.^31^

### Statistical Analyses

The results were presented as the mean ± standard deviation. Statistical significance was assessed using analysis of variance utilizing IBM SPSS Statistics version 20.0 software, and the letter notation method was employed for indicating significant differences in multiple comparisons. Differences denoted by distinct letters were indicative of significant variations (*p* < 0.05). Graphical representations were generated using GraphPad Prism version 6.01.

## Results and Discussion

### TLC Analysis of Octacosanol Lipoate

**Figure 1:**
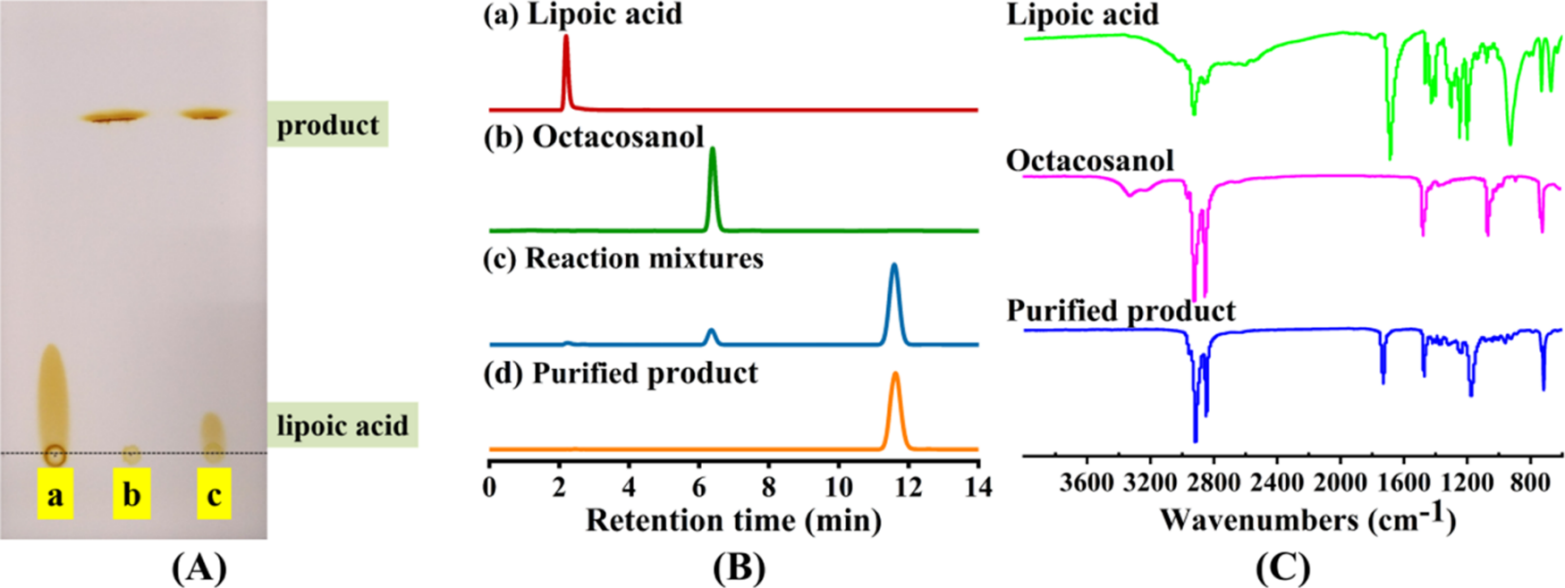
(A) Thin-layer chromatography analysis: From left to right: a. reaction substrate, b. purified product (octacosanol lipoate), c. reaction sample. (B) HPLC analysis results. From top to bottom: *a*. lipoic acid, *b*. octacosanol, *c*. reaction mixture, *d*. purified product (octacosanol lipoate). (C) FT-IR spectra results. From top to bottom: lipoic acid, octacosanol, and purified product (octacosanol lipoate).

Pre-experiment results showed that octacosanol does not exhibit any color in iodine vapor. As shown in Figure 1A, the *R*_f_ value of lipoic acid in the mixed substrates (left sample) ranged from 0 to 0.375. In contrast, the reaction mixture (right sample) showed a new spot at an *R*_f_ value of 0.875, indicating the formation of a new compound. This new compound had a significantly different *R*_f_ value from that of lipoic acid, indicating a substantial difference in polarity between the two and facilitating easy separation. The middle sample represented the product obtained after purification on a silica gel column, where only the spot corresponding to the new compound was present, suggesting the removal of lipoic acid from the reaction mixtures.

### HPLC Analysis of Octacosanol Lipoate

Lipoic acid (curve *a*) and octacosanol (curve *b*) were eluted at a retention time of 2.21 and 6.39 min, respectively (Figure 1B). In the reaction mixture (curve *c*), a new peak emerged at 11.29 min, indicating the presence of a newly formed substance distinct from the two substrates. Curve *d* represented the purified product obtained through silica gel column chromatography, which was devoid of the two substrates and exhibited a purity exceeding 99%. This suggested that the chosen conditions for silica gel column chromatography effectively separate the new substance.

### FT-IR Spectroscopy of Octacosanol Lipoate

Figure 1C displays the FT-IR spectra of lipoic acid (top curve), octacosanol (middle curve), and octacosanol lipoate (the bottom curve). In lipoic acid (the top curve), the broad peak below 2100–3500 cm^–1^ and the strong peak at 1688.47 cm^–1^, respectively, corresponded to the characteristic absorption signals of the hydroxyl group (−OH) and the carbonyl group (–C=O−) within the carboxyl group (−COOH). The characteristic absorption signal of the hydroxyl group (−OH) in octacosanol (the middle curve) was detected at 3321.26 cm^–1^. In the FT-IR spectrum of the product (the bottom curve), the absorption signals of the carboxyl group (COOH) in lipoic acid and the free hydroxyl group (−OH) in octacosanol vanished, whereas new absorption peaks appeared at 1728.75 and 1175.12 cm^–1^, which corresponded to the characteristic absorption signals of –C=O and C–O within the ester bond, respectively. Thus, the synthesized substance was proven to be octacosanol lipoate.

### HPLC-MS Analysis of Octacosanol Lipoate

**Figure 2:**
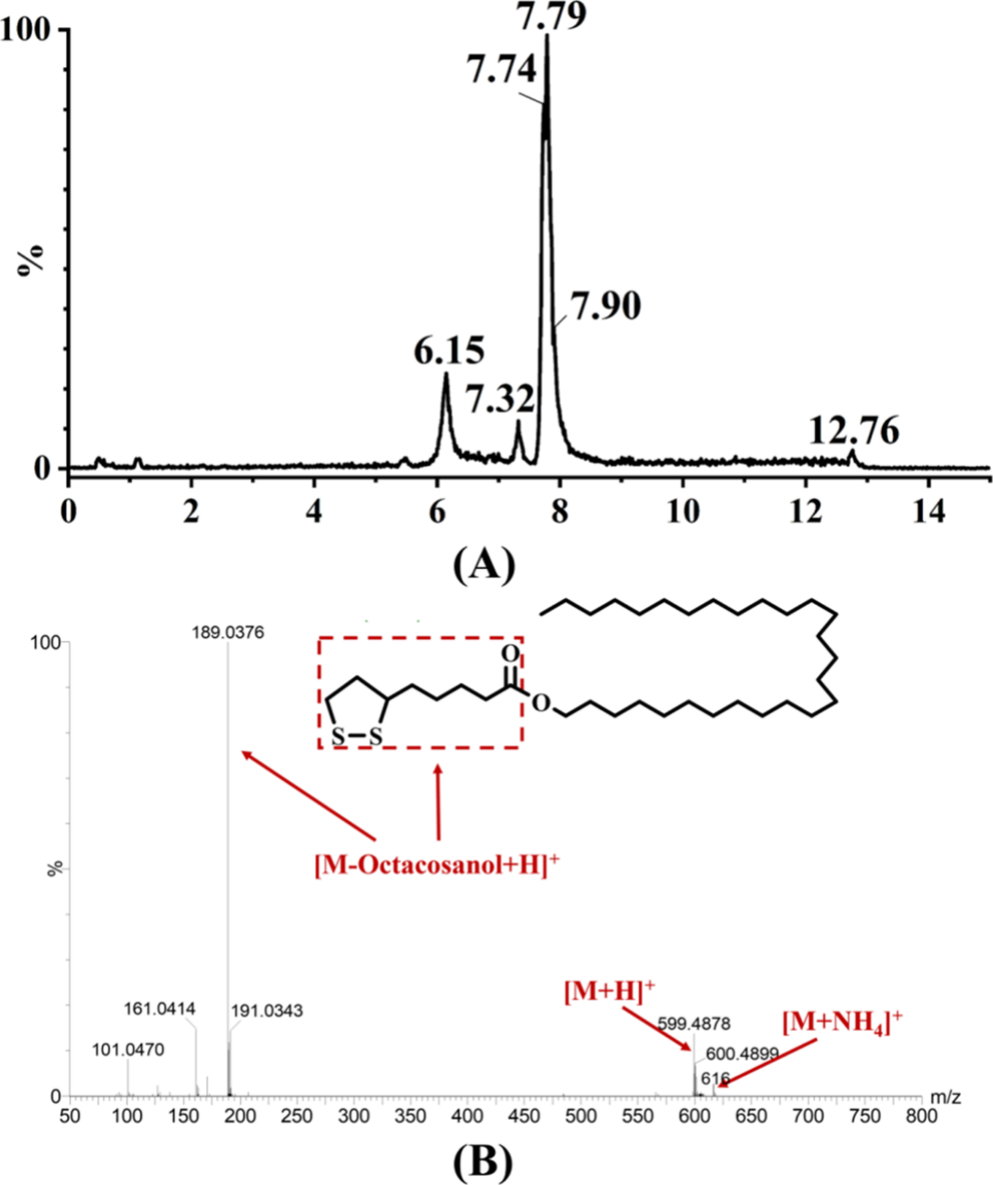
HPLC-MS chromatogram of octacosanol lipoate. (A) Total ion flow diagram of the product; (B) mass spectra of the sample at retention time 7.79 min.

Figure 2A,B depicts the total ion chromatogram of the reaction products and the mass spectrum of the chromatographic peak at a retention time of 7.79 min, respectively. The theoretical molecular weight of octacosanol lipoate was 598. In Figure 2B, signals were detected at mass-to-charge ratios (*m*/*z*) of 599, 616, and 189, corresponding to [M + H]^+^, [M + NH_4_]^+^, and [M-octacosanol + H]^+^, respectively. This indicated that the synthesized product was octacosanol lipoate.

### NMR Analysis of Octacosanol Lipoate

**Figure 3:**
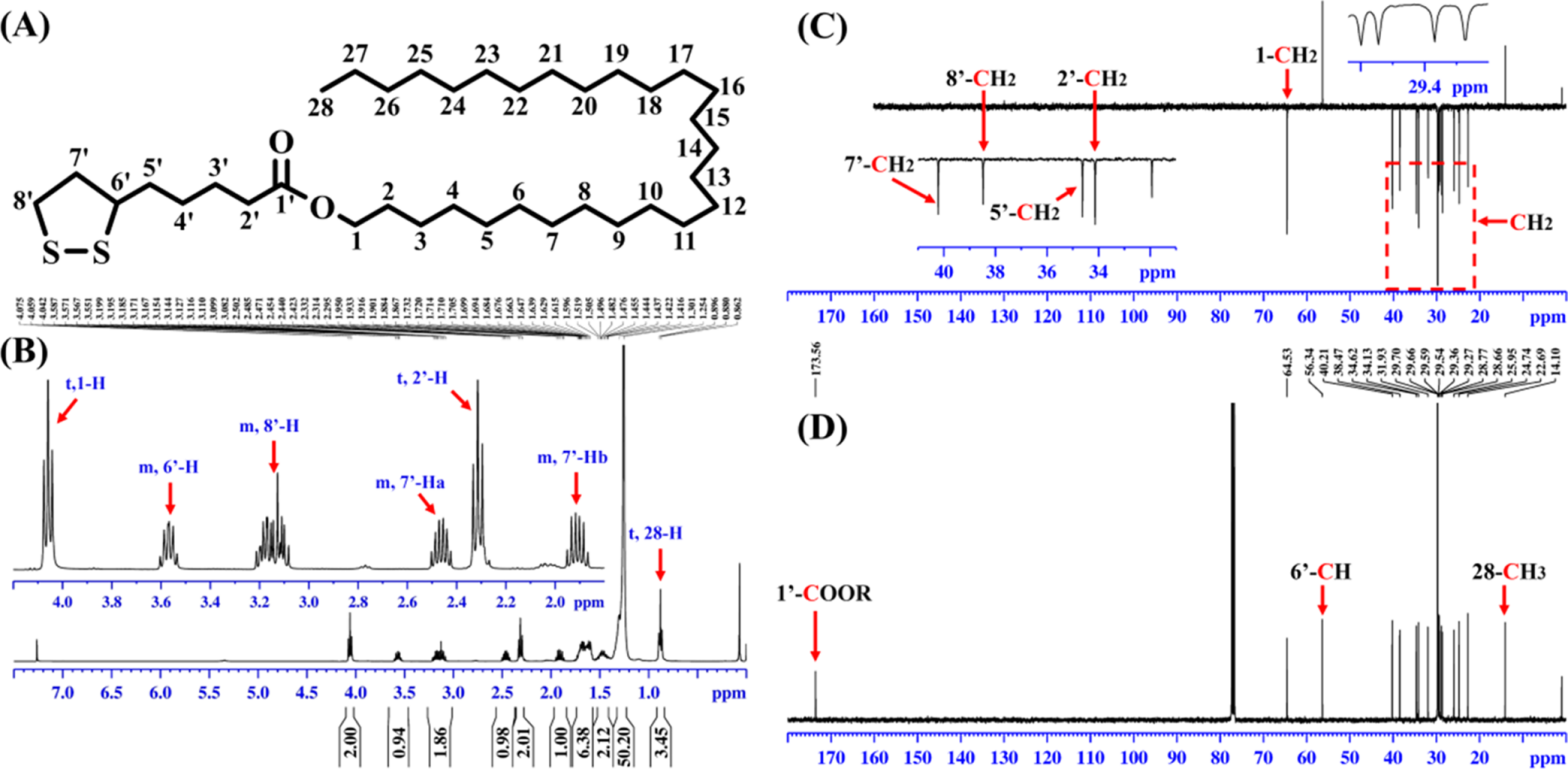
(A) Chemical structure of octacosanol lipoate and (B) ^1^H NMR, (C) DEPT-135, and (D) ^13^C NMR spectra of octacosanol lipoate.

The chemical structure of the target product and its corresponding NMR spectra are presented in Figure 3. In the ^1^H spectrum (Figure 3B), there were many overlapping absorption signals in the range of 1.25–1.31 ppm, corresponding to the numerous −CH_2_ groups in the octacosanol moiety of the product molecule, as these H atoms had similar chemical environments. The triplet peak at 0.88 ppm corresponded to the absorption signal of the 28-H. In the octacosanol, the resonance absorption signal of 1-H was at 3.60 ppm,^32^ while in the product molecule, the chemical shift of 1-H was increased by 0.46 ppm, shifting to 4.06 ppm, indicating the presence of an ester bond in the product. Due to the inductive effect of the ester bond, the electron cloud density around the hydrogen atoms was reduced, resulting in the chemical shift moving to a lower field. Similarly, in the octacosanol linoleate, the chemical shift of 1-H moved to 4.05 ppm.^22^ According to the previous studies,^33,16^ the resonance absorption signals of the 6′-, 7′-, and 8′-H of the lipoic acid molecule in the product emerged at 3.56, 2.45 (7′-H_a_), 1.90 (7′-H_b_), and 3.12 ppm, respectively. Furthermore, the integration areas of the hydrogen atoms in the product were consistent with the number of hydrogen atoms in octacosanol lipoate.

All of the signals of all carbon atoms in the product are present in the ^13^C NMR spectrum (Figure 3D). In the DEPT-135 spectrum, the resonances of methylene (CH_2_) carbons are negative peaks, while methyl (CH_3_) and tertiary (CH) carbons are positive peaks, and quaternary carbons do not show any absorption signals (Figure 3C). The absorption signal at 173.56 ppm appeared in the ^13^C NMR spectrum but was absent in the DEPT-135 spectrum, corresponding to the 1′-COOR of the product, consistent with the predicted chemical structure of octacosanol lipoate. The downward peaks between 22.5 and 40.5 ppm can be attributed to CH_2_ (Figure 3C). The peak at 14.10 ppm in the ^13^C NMR spectrum corresponded to 28-CH_3_, the peak at 56.34 ppm corresponded to 6′-CH, and the peak at 64.53 ppm corresponded to 1-CH_2_. Compared to the signal of octacosanol (63.0 ppm),^32^ the signal of 1-C has shifted downfield by 1.53 ppm, indicating that 1-C was located at the ester linkage. This was consistent with the 1-C signal peak of octacosanol lipoate.^22^ In conclusion, these results, in conjunction with TLC, HPLC, FT-IR, and HPLC-MS findings, demonstrated the successful synthesis of the target product, octacosanol lipoate, using a lipase-catalyzed route.

### Screening of Lipase

**Figure 4:**
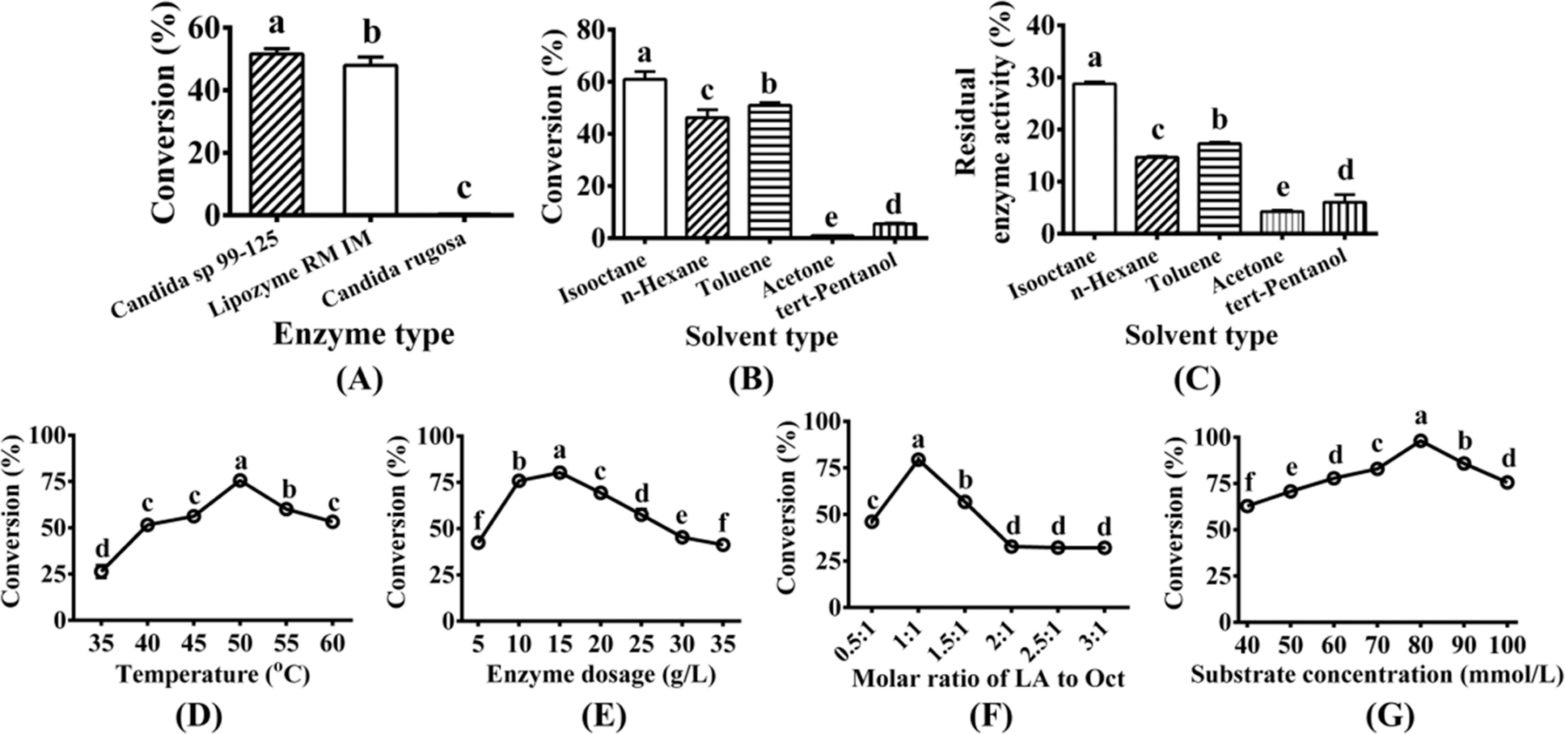
Effect of different reaction parameters on product conversion. (A) Enzyme type: 50 mmol/L lipoic acid, 1:1 molar ratio of lipoic acid to octacosanol, 10 g/L lipase, 120 g/L molecular sieves, isooctane, 40 °C, 2h. (B) Solvent type: 50 mmol/L lipoic acid, 1:1 molar ratio of lipoic acid to octacosanol, 10 g/L *Candida sp*. 99–125, 120 g/L molecular sieves, 40 °C, 2 h. (C) Effect of solvent type on residual enzyme activity. (D) Reaction temperature: 50 mmol/L lipoic acid, 1:1 molar ratio of lipoic acid to octacosanol, 10 g/L *Candida sp*. 99–125, 120 g/L molecular sieves, isooctane, 2 h. (E) Lipase dose: 50 mmol/L lipoic acid, 1:1 molar ratio of lipoic acid to octacosanol, *Candida sp*. 99–125, 120 g/L molecular sieve, isooctane, 50 °C, 2 h. (F) Molar ratio of lipoic acid to octacosanol: 50 mmol/L lipoic acid, 15 g/L *Candida sp*. 99–125, 120 g/L molecular sieve, isooctane, 50 °C, 2 h. (G) Concentration of lipoic acid: 1:1 molar ratio of lipoic acid to octacosanol, 15 g/L *Candida sp*. 99- 125 lipase, 120 g/L molecular sieves, isooctane, 50 °C, 2 h. Data labeled with different lowercase letters indicate significant differences (*p* < 0.05).

The effects of three lipases, namely, *Candida rugosa*, *Candida sp*. 99–125, and Lipozyme RM IM, on the esterification of lipoic acid and octacosanol were investigated. Figure 4A demonstrates that when *C. rugosa* was employed as the catalyst, the conversion rate was merely 0.4%, resulting in negligible product formation. On the other hand, *Candida sp.* 99–125 exhibited the highest catalytic performance among the lipases, achieving a conversion rate of approximately 51%. Lipozyme RM IM displayed a slightly lower catalytic performance at 50%. *Candida sp.* 99–125 lipase possesses several advantages, including low cost, high enzyme activity, and good stability, making it widely utilized in the synthesis of alkyl oleate,^34^ ergosterol linolenate,^35^ ε-caprolactone,^36^ xylitol esters,^37^ and other compounds. Notably, there was currently no research exploring the use of *Candida sp.* 99–125 lipase for the esterification of lipoic acid, making it an attractive candidate for further investigation.

### Reaction Solvent

The choice of reaction solvent was of the utmost importance in the lipase-catalyzed esterification process. This study investigated the effects of five organic solvents, namely, isooctane, *n*-hexane, toluene, acetone, and *tert*-pentanol, on the esterification of lipoic acid and octacosanol (Figure 4B). Remarkably, when isooctane was employed as the solvent under identical reaction conditions, the conversion rate reached an impressive 60.9%. Toluene and *n*-hexane closely followed suit, exhibiting relatively high conversion rates. Conversely, the use of acetone and *tert*-pentanol as solvents resulted in considerably lower conversion rates, with *tert*-pentanol yielding a mere 5.5% and acetone barely producing any product. Several factors may account for these findings. First, the choice of reaction solvent significantly influences the reaction’s progression by altering the solubility of the substrate. In the case of isooctane, the substrate gradually dissolves over time, facilitating a dynamic equilibrium process for the reaction to proceed. On the other hand, the enzyme’s activity and stability are positively correlated with the hydrophobicity of the organic solvent used.^38^ Hydrophobicity is often quantified using log *P* values, where higher values indicate greater hydrophobicity. In this study, isooctane, *n*-hexane, toluene, acetone, and *tert*-pentanol have log *P* values of 4.70, 3.50, 3.83, −0.23, and 1.30, respectively.^23,39^ Among these solvents, isooctane exhibits the highest log *P* value, indicating its strongest hydrophobicity and the least ability to remove the “essential water” from the enzyme’s active center. Consequently, isooctane yields the highest conversion rate. Similarly, the relatively high log *P* values of *n*-hexane and toluene were associated with their high conversion rates. In contrast, the low log *P* values of acetone and *tert*-pentanol suggested weak hydrophobicity, which inhibited enzyme activity by removing essential water, resulting in lower conversion rates. To further assess the stability of the lipase, its residual activity was measured after treatment with different solvents under identical conditions (Figure 4C). The highest residual enzyme activity was observed after treatment with isooctane, reaching 28.8%, indicating superior stability in this solvent. Slightly lower residual enzyme activities were observed with toluene and *n*-hexane, while *tert*-pentanol and acetone significantly inhibited lipase activity, rendering it almost inactive. Similarly, isooctane was identified as the optimal reaction medium for synthesizing phytoterol lipoate and ergosterol linolenate using *Candida sp*. 99–125 lipase.^16,35^ Therefore, isooctane was selected as the preferred reaction solvent.

### Reaction Temperature

The impact of reaction temperature on the esterification of lipoic acid and octacosanol was investigated within the temperature range of 35–60 °C (Figure 4D). Within the range of 35–50 °C, the conversion rate exhibited a gradual increase with rising temperature. At 35 °C, the conversion rate was merely 26.4%, whereas at 50 °C, it reached 75.7%. This can be attributed to the enhanced solubility of the substrate in the solvent, resulting from the elevated reaction temperature, which in turn accelerated mass transfer reactions. However, as the temperature continued to rise, the conversion rate displayed a linear decline. When the reaction temperature reached 60 °C, the conversion rate dropped to 53.3%. This reduction in conversion rate can be attributed to the progressive deactivation of the lipase enzyme as it surpasses its optimal temperature. Zhao et al. discovered that *Candida sp*. 99–125 facilitated the synthesis of monoglycerides at an optimal reaction temperature of 50 °C.^40^ Therefore, in this study, 50 °C was selected as the optimum temperature.

### Lipase Dose

The impact of *Candida sp*. 99–125 lipase dosage on the conversion rate was investigated (Figure 4E). At a lipase dosage of 5 g/L, the conversion rate was 42.5%. As the lipase dosage increased, the conversion rate gradually rose and reached 80.3% at 15 g/L. However, a further increase in enzyme dosage resulted in a decrease in the conversion rate, with a rate of 41.3% observed at a lipase dosage of 35 g/L. This decline may be attributed to excessive lipase hindering effective contact between lipoic acid and octacosanol, impeding mass transfer between the two compounds, and ultimately reducing the conversion rate. Therefore, a dosage of 15 g/L of *Candida sp*. 99–125 lipase was considered optimal.

### Substrate Molar Ratio

The impact of the molar ratio between lipoic acid and octacosanol ranging from 0.5:1 to 3:1 on the conversion rate was investigated. Esterification reactions are usually reversible, and increasing the quantity of one substrate can enhance the reaction toward esterification.^35^ As depicted in Figure 4F, when the molar ratio of lipoic acid to octacosanol increased from 0.5:1 to 1:1, the conversion rate exhibited a rapid rise from 46.1 to 79.4%. However, a further increase in lipoic acid led to a significant decline in the conversion rate, reaching a low rate of 32.8% at a molar ratio of 2:1. This may be attributed to the excess lipoic acid causing the reaction system to become acidic, partially inhibiting the lipase activity and thus suppressing esterification. Further increasing the acid-to-alcohol molar ratio did not show any significant changes in the conversion rate. Zhong et al. also discovered that the optimal molar ratio for the esterification reaction of oleic acid and alkyl alcohols catalyzed by *Candida sp*. 99–125 lipase was 1:1.^34^ Therefore, a molar ratio of 1:1 for lipoic acid/octacosanol was chosen for subsequent experiments.

### Substrate Concentration

The impact of lipoic acid concentration on the conversion rate was investigated in the range of 40–100 mmol/L. As depicted in Figure 4G, at a lipoic acid concentration of 40 mmol/L, the conversion rate reached 62.9%. Increasing the concentration from 40 to 80 mmol/L significantly enhanced the conversion rate, peaking at 98.1% at 80 mmol/L. This can be attributed to the increased likelihood of molecular contact with the rise in substrate concentration. However, surpassing 80 mmol/L lipoic acid concentration resulted in a linear decline in the conversion rate, with a mere 75.7% at a concentration of 100 mmol/L. This can be attributed to two factors. First, there is a limited capacity for substrate dissolution, and only dissolved substrate can partake in the esterification reaction. Second, the lipase enzyme’s active sites are limited, and when the substrate concentration exceeds the lipase’s catalytic threshold, it becomes insufficient to catalyze the esterification reaction.^23^ Consequently, a lipoic acid concentration of 80 mmol/L was selected.

### Product Yield

To verify the viability of the optimal parameters obtained from this study, octacosanol lipoate was synthesized under the following optimized conditions: 80 mmol/L lipoic acid and 15g/L *Candida sp*. 99–125 lipase, a 1:1 molar ratio of lipoic acid to octacosanol, and 50 °C, attaining a conversion rate of over 95%. The yield of the final product was determined through silica gel column chromatography. The results showed that the actual yield of octacosanol lipoate was 87.9%, marginally lower than the conversion rate. This disparity can be ascribed to the unavoidable loss of a small amount of product during the column chromatography separation process. As a result, the optimal process parameters acquired in this study were deemed feasible.

### Melting Point of Octacosanol Lipoate and Its Lipophilicity

**Figure 5:**
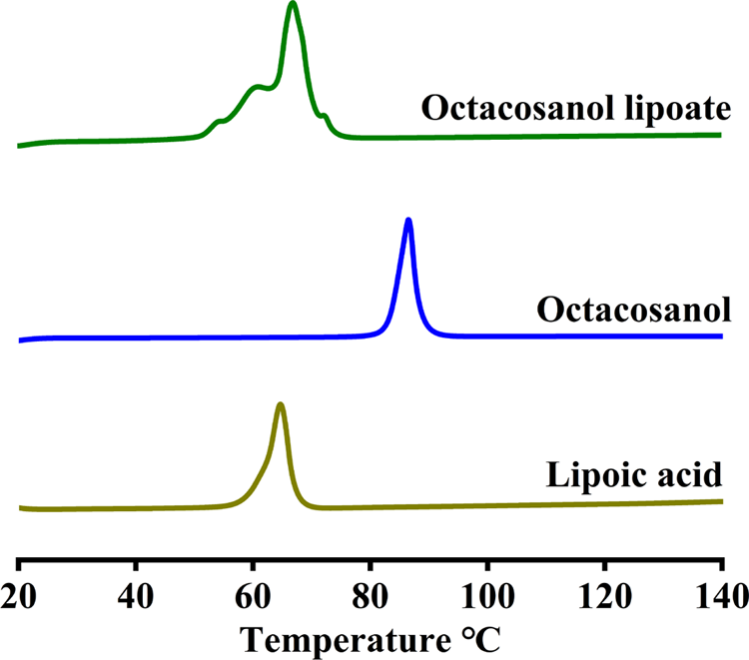
Differential scanning calorimetry analysis of octacosanol lipoate, octacosanol, and lipoic acid.

The melting point of lipoic acid was approximately 65 °C, while that of octacosanol was nearly 90 °C. Nonetheless, the melting point of octacosanol lipoate ranged from 70 to 80 °C, situating itself between the two (Figure 5). This suggested that the esterification process enhanced the thermal stability of lipoic acid and facilitated the melting of octacosanol. Commonly, a higher log *P* value signifies stronger lipophilicity. With a log *P* value of 2.04, lipoic acid became more lipophilic after esterification, as evidenced by a log *P* value of 14.17. This significant enhancement in lipophilicity expanded the application scope of lipoic acid in oils and fats.

### POV, *p*-AnV, and TOTOX Values

**Figure 6:**
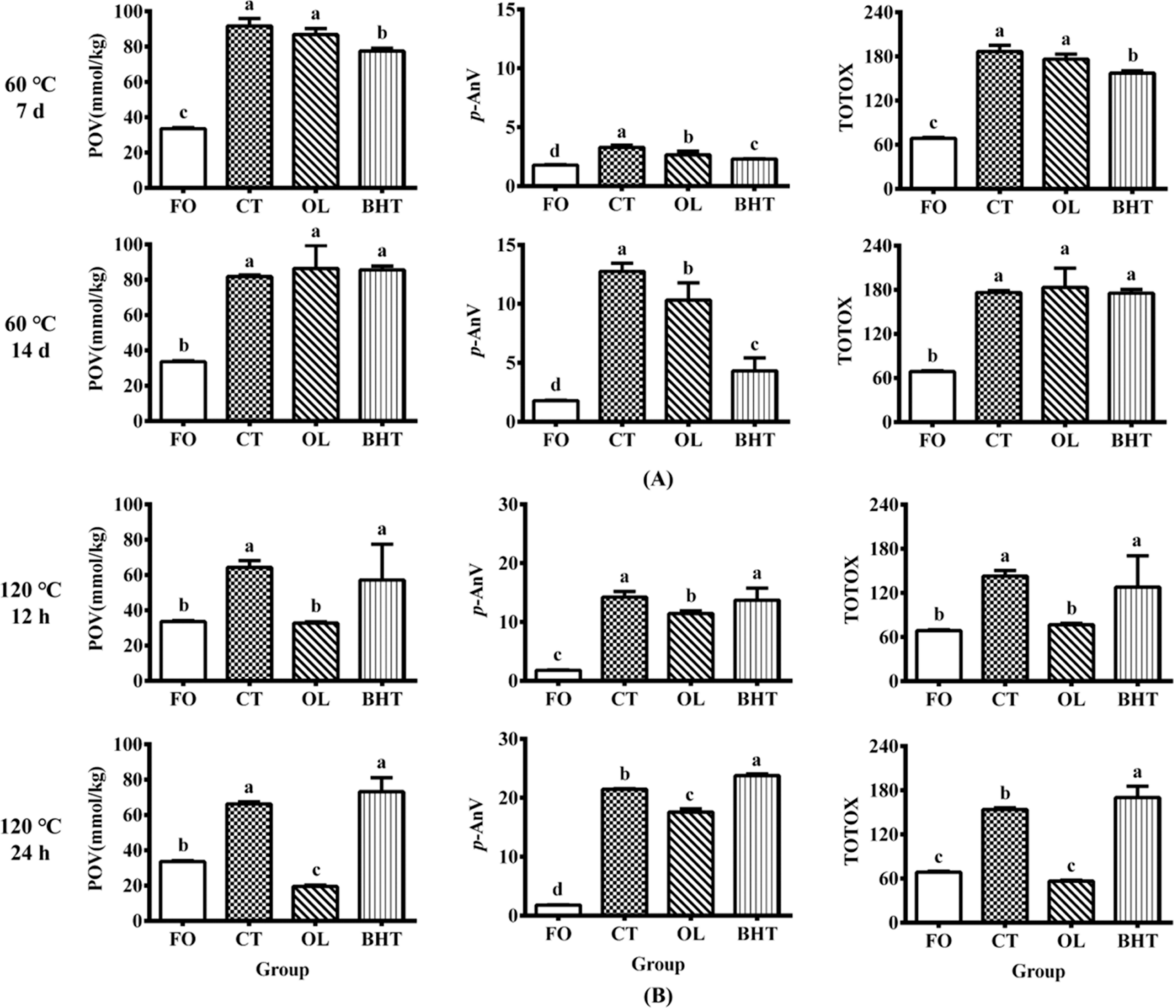
POV, *p*-AnV, and TOTOX values of sunflower oil after heating at (A) 60 °C and (B) 120 °C. **FO**: fresh unheated sunflower oil; **CT**: control sunflower oil; **BHT**: sunflower oil with addition of 200 ppm butylated hydroxytoluene; **OL**: sunflower oil with addition of an equimolar amount of octacosanol lipoate.

The degree of lipid oxidation is generally assessed by several indicators such as POV and *p*-AnV, which reflect the content of primary oxidation products (hydroperoxides) and secondary oxidation products (aldehydes, ketones, etc.), respectively. Nevertheless, lipid oxidation is a complex and dynamic process, and POV or *p*-AnV alone cannot fully reflect the true oxidation status. TOTOX or integral oxidation value calculated as the sum of POV and *p*-AnV can indicate the oxidation status of lipids to a certain extent.^41^ In this study, we measured the POV, *p*-AnV, and TOTOX values of sunflower oil with different treatments. As shown in Figure 6, the POV, *p*-AnV, and TOTOX of fresh unheated sunflower oil (FO) were 33.51, 1.78, and 68.79 mmol kg^–1^, respectively. Upon subjecting the oil to 60 and 120 °C, the POV, *p*-AnV, and TOTOX values of the control group (CT) significantly rose, in comparison to FO, indicating that heating accelerated the oxidative rancidity of sunflower oil. Although OL did not significantly reduce the POV and TOTOX values compared to the CT group after 7 and 14 days of heating, it did significantly reduce the *p*-AnV values by 18.77 and 19.17%, respectively. In contrast, BHT showed a significant decrease in POV, *p*-AnV, and TOTOX values, indicating that the antioxidant activity of OL at 60 °C was not as good as that of BHT.

The POV of CT increased to 64.30 and 66.24 mmol kg^–1^ after 12 and 24 h of heating at 120 °C, respectively, with an increase of 91.91 and 97.70%, respectively, compared to FO. Meanwhile, the addition of OL led to a decrease in the POV values of the CT group by 49.20 and 70.68%, respectively, demonstrating significant antioxidant efficacy. However, there was no significant decrease in the BHT group when compared with the CT group under the same conditions. A similar trend was also observed in terms of *p*-AnV and TOTOX after 12 and 24 h of treatment at 120 °C. Specifically, after heating at 120 °C for 12 h, OL markedly diminished the *p*-AnV and TOTOX values by 19.53 and 46.24%, respectively, compared to the CT group, while at 24 h, this reduction reached 18.06 and 63.34%, respectively. In contrast, the addition of BHT failed to yield a notable decrease in these indices. These findings suggested that OL exhibited a substantially superior antioxidant capacity to BHT under conditions of high temperature. It was noteworthy that the *p*-AnV and TOTOX values of the BHT group instead increased compared to those of the CT group after heating at 120 °C for 24 h. This may be due to the fact that BHT is less stable at high temperatures and undergoes oxidation reactions itself. A similar situation was found in the study by Reda that BHT started to decompose at 120 °C.^42^ The oxidation process of oils and fats is a complex dynamic process, i.e., primary oxidation products will be decomposed into secondary and tertiary oxidation products sequentially.^43^ Although the content of tertiary oxidation products was not measured in this study, the available results suggest that the antioxidant effect of OL may be inferior to that of BHT at 60 °C, but the antioxidant properties of OL significantly outperform those of BHT at a high temperature of 120 °C.

### Feasibility of Determining the Oxidation of Oils and Fats by ^1^H NMR

**Figure 7:**
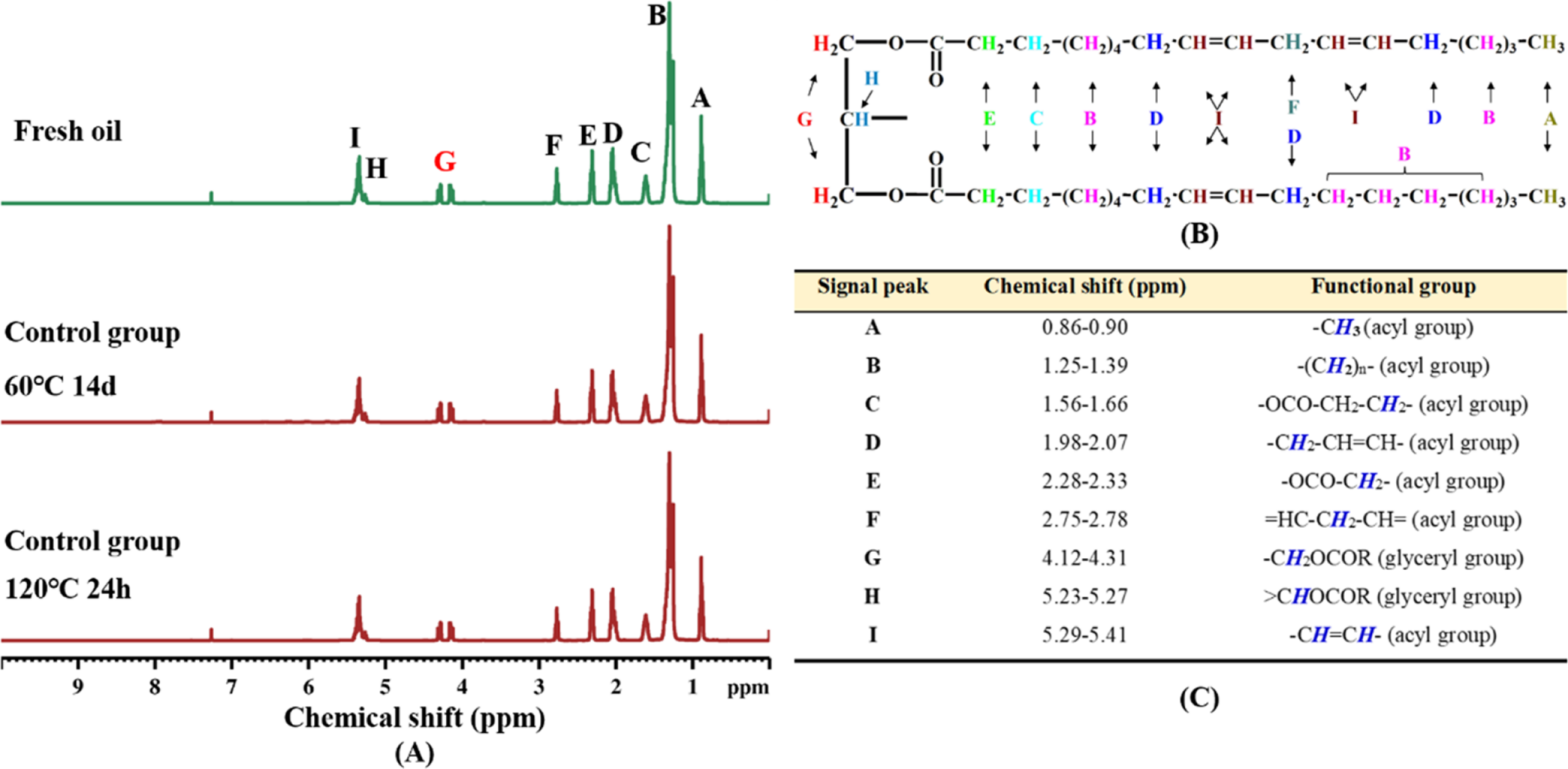
^1^H NMR spectra (A) of sunflower oils (fresh, 60 °C 14d, 120 °C 24 h), their typical fatty acid glyceride structure (B), and the attribution of major hydrogen proton in the ^1^H NMR spectra of sunflower oils (C). **FO**: fresh unheated sunflower oil; **CT**: control sunflower oil; **BHT**: sunflower oil with addition of 200 ppm butylated hydroxytoluene; **OL**: sunflower oil with addition of an equimolar amount of octacosanol lipoate.

Sunflower oil contains primarily triacylglycerols consisting of linoleic acid and oleic acid. The ^1^H NMR spectra of fresh and heat-treated sunflower oils and their typical triacylglycerol structure are shown in Figure 7. Nine groups of signal peaks with varying intensities were observed in the chemical shift range 0–5.5 ppm, and the peak intensity was proportional to the content of H protons in the respective chemical environments. Visually, the H proton signals in the sunflower oil showed no perceptible changes after heat treatment. This can be ascribed to the fact that, at temperatures of 60 and 120 °C, the quantity of oil that underwent oxidation constituted a relatively small portion of the total oil, thus having a negligible impact on the overall H proton signal within the oil.

**Figure 8:**
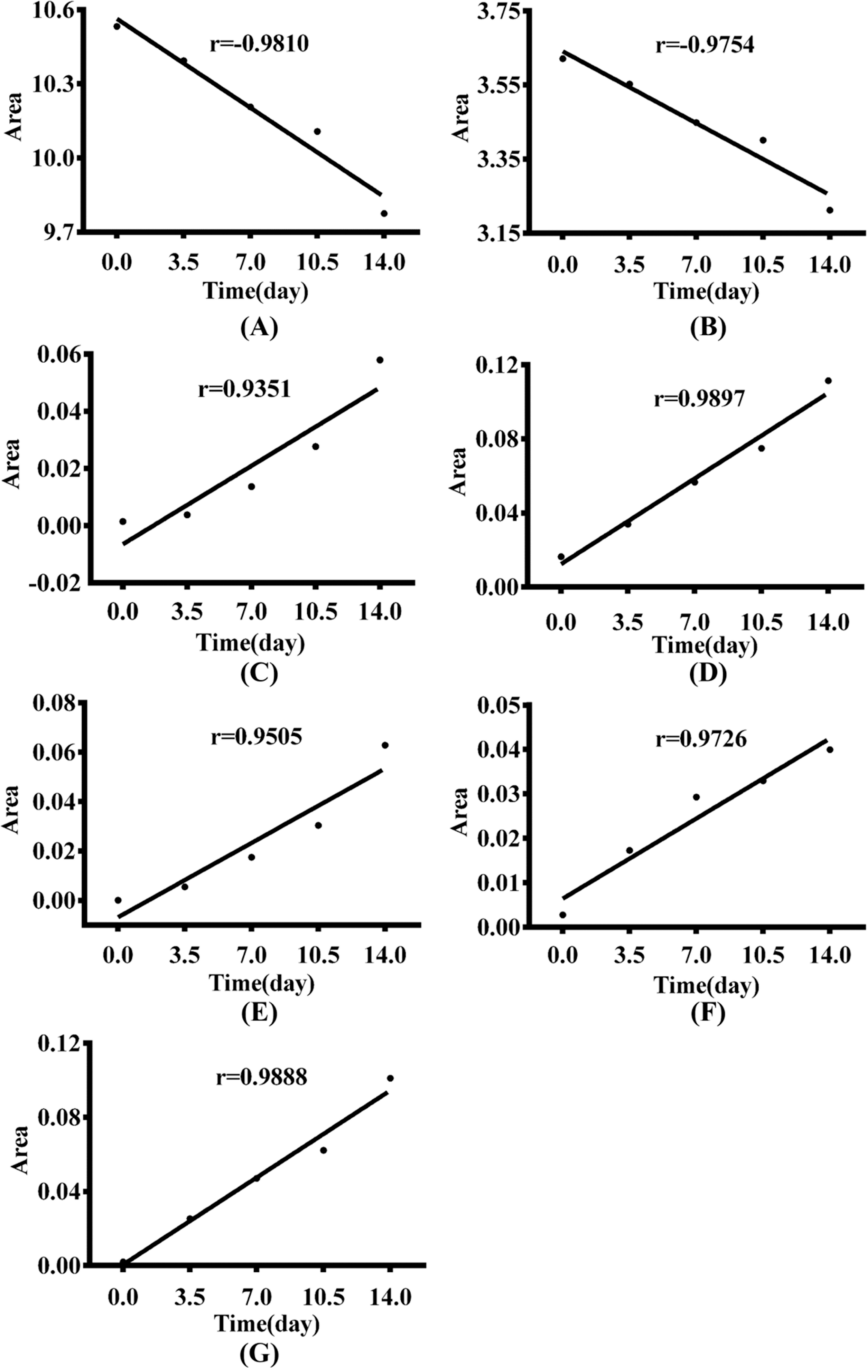
Fitted curves between different treatment times and integral areas at different chemical shifts for sunflower oil treated at 60 °C: (A) 1.90–2.15 ppm, (B) 2.70–2.85 ppm, (C) 5.71–5.78 ppm, (D) 5.95–6.1 ppm, (E) 6.215–6.305 ppm, (F) 6.52–6.62 ppm, (G) 7.9–8.06 ppm.

The degree of lipid oxidation can be assessed by the decrease in intensity of the characteristic H proton signals of main fatty acids and the increase in intensity of the characteristic H signals of oxidation products in the ^1^H NMR. Among the numerous H protons in triacylglycerols, the CH_2_ proton signal of the glycerol backbone has a higher stability, with a chemical shift at 4.0–4.4 ppm, and it does not overlap with other H proton signals.^44^ Therefore, in this study, the CH_2_ proton signal of the glycerol backbone (4.0 and 4.4 ppm) was selected as the integration benchmark. The chemical shifts 1.90–2.15 ppm and 2.70–2.85 ppm corresponded to the signals of allylic and diallylic H protons in unsaturated fatty acids, and their integration values indicated the content of unsaturated fatty acids. As shown in Figure 8A,B, with the extension of heating time, the integration intensity of 1.90–2.15 and 2.70–2.85 ppm in sunflower oil decreased linearly, with absolute value of the correlation coefficient (*r* values) greater than 0.97. Additionally, the chemical shifts 5.7–6.7 and 7.9–8.06 ppm represented the characteristic H protons in secondary and primary oxidation products, respectively, and their integration values reflected the content of these oxidation products. As shown in Figure 8C–G, with the extension of heating time, the integration intensity of 5.71–5.78, 5.95–6.1, 6.215–6.305, 6.52–6.62, and 7.9–8.06 ppm in sunflower oil increased linearly, with *r* values greater than 0.93. This suggested that using the CH_2_ proton signal of the glycerol backbone (4.0–4.4 ppm) as a reference for integrating the characteristic H protons in main fatty acids and oxidation products can accurately and reliably reflect the degree of oil oxidation.

### Inhibition of Major Fatty Acid Degradation in Sunflower Oil by Octacosanol Lipoate

**Figure 9:**
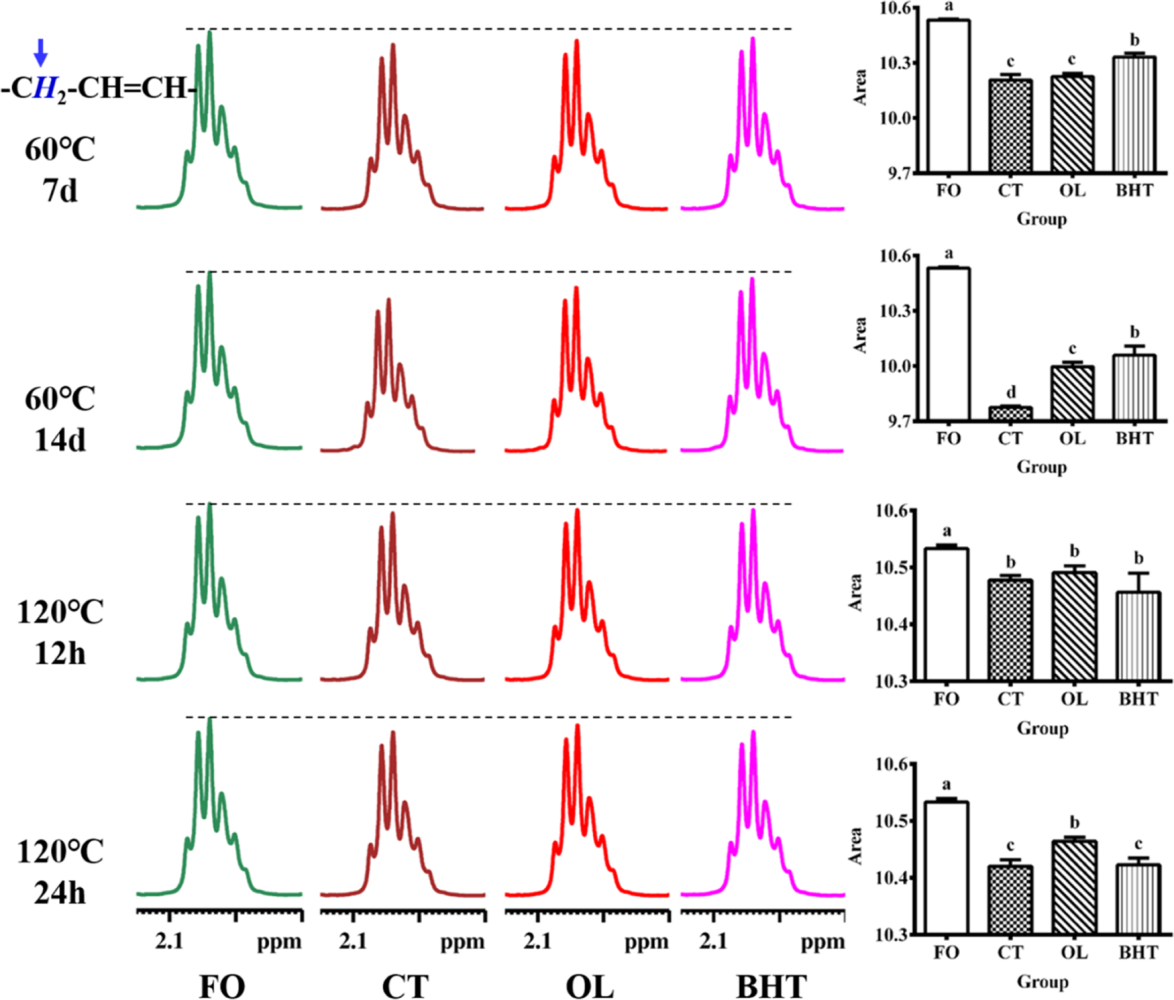
Amplified ^1^H NMR spectra of sunflower oil at different temperatures (60 °C, 120 °C) at chemical shift of 1.90–2.15 ppm. **FO**: fresh unheated sunflower oil; **CT**: control sunflower oil; **BHT**: sunflower oil with addition of 200 ppm butylated hydroxytoluene; **OL**: sunflower oil with addition of an equimolar amount of octacosanol lipoate.

**Figure 10:**
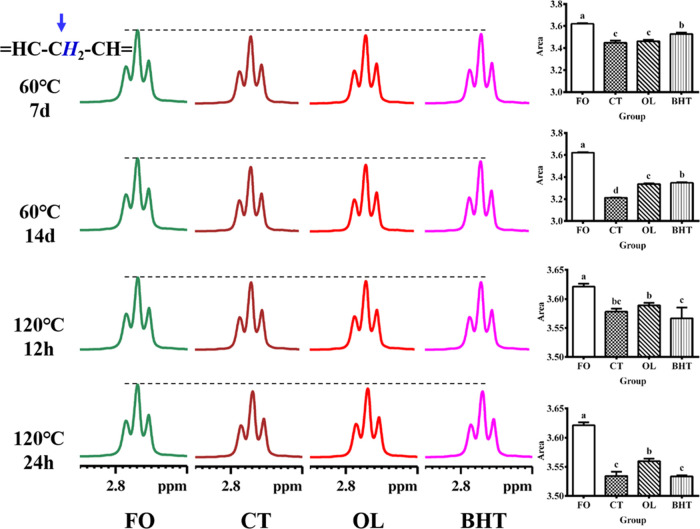
Amplified ^1^H NMR spectra of sunflower oil at different temperatures (60 °C, 120 °C) at chemical shift of 2.70–2.85 ppm. **FO**: fresh unheated sunflower oil; **CT**: control sunflower oil; **BHT**: sunflower oil with addition of 200 ppm butylated hydroxytoluene; **OL**: sunflower oil with addition of an equimolar amount of octacosanol lipoate.

Linoleic and oleic acids are the predominant fatty acids in sunflower oil and are highly prone to oxidation upon heating. The proton signal at 1.90 and 2.15 ppm pertains to the allylidene methylene group (–C***H***_2_–CH=CH−) in both linoleic and oleic acids. The proton signal at 2.70–2.85 ppm was that of the bis-allylidene methylene group (=HC–C***H***_2_–CH=) in linoleic acid. As shown in Figures 9 and 10, the proton signals of both allyl and bisallyl progressively decreased with the heating time, indicating that both oleic and linoleic acids in sunflower oils were significantly degraded. The lowest proton signal intensities for allyl and diallyl were observed when heated at 60 °C for 14 days, meaning that the degradation was most severe under this heating condition. The proton signal intensities at 1.90–2.15 and 2.70–2.85 ppm were reduced by 7.19 and 11.29%, respectively, in the CT group as compared to FO. The signal intensity increased by 2.26 and 3.87% in the OL group and by 2.90 and 4.22% in the BHT group, respectively, as compared to the CT group. This suggested that both OL and BHT could suppress the oxidative degradation of unsaturated fatty acids, and the inhibitory effect of BHT was significantly superior to that of OL. However, after heating at 120 °C for 24 h, there was no significant difference in the allyl and diallyl signals between the BHT and CT groups, but there was a significant increase in the OL group compared with the CT group, suggesting that OL could effectively inhibit the degradation of unsaturated fatty acids induced by high temperatures, whereas BHT did not have such an effect.

### Inhibition of Oxidation Product Formation by Octacosanol Lipoate

**Figure 11:**
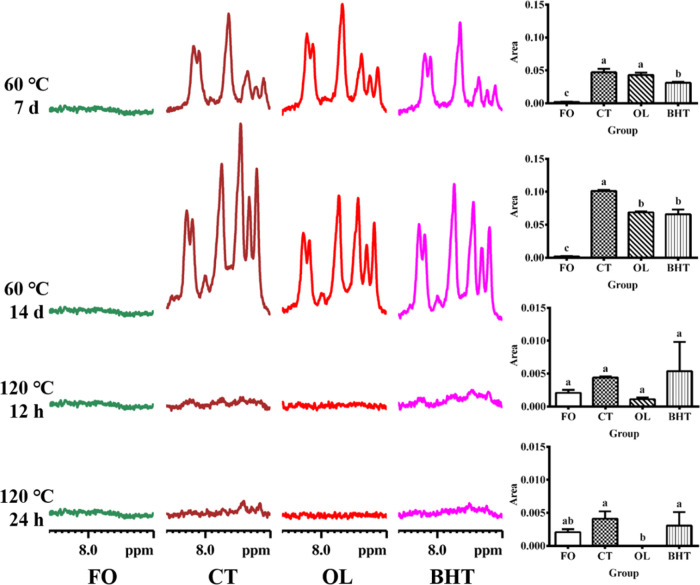
Amplified ^1^H NMR spectra of sunflower oil at different temperatures (60 °C, 120 °C) at chemical shift of 7.9–8.06 ppm. **FO**: fresh unheated sunflower oil; **CT**: control sunflower oil; **BHT**: sunflower oil with addition of 200 ppm butylated hydroxytoluene; **OL**: sunflower oil with addition of an equimolar amount of octacosanol lipoate.

Hydrogen peroxides are the primary oxidation products in the oxidation process of oils, and their characteristic signals mainly emerge around 8.0 ppm. As depicted in Figure 11, the signal of hydroperoxides scarcely manifested in FO, but upon heating at 60 °C, a notable signal emerged in the range 7.9–8.06 ppm, and the intensity of the signal progressively increased with the elongation of the oxidation time, indicating that hydroperoxides were generated after heating at this temperature. When heated at 60 °C for 14 days, the addition of both OL and BHT significantly diminished the signal intensity of hydroperoxides as compared to the CT group, and the signal intensities of OL and BHT decreased the peroxides by 31.97 and 34.84%, respectively, compared to the CT group, and there was no significant difference between them, suggesting that OL could suppress the production of hydroperoxides induced by prolonged heating at low temperatures, and the effect was comparable to that of BHT. However, after heating at 120 °C for 12 and 24 h, there was no significant difference in the signal intensity of hydrogen peroxides in the CT group compared to FO. This was in line with the results observed in the ^1^H NMR spectra of soybean oil heated at 180 °C.^44^ This might be due to the fact that primary oxidation products are typically unstable and further decompose into secondary oxidation products, such as small molecule aldehydes and ketones.

**Figure 12:**
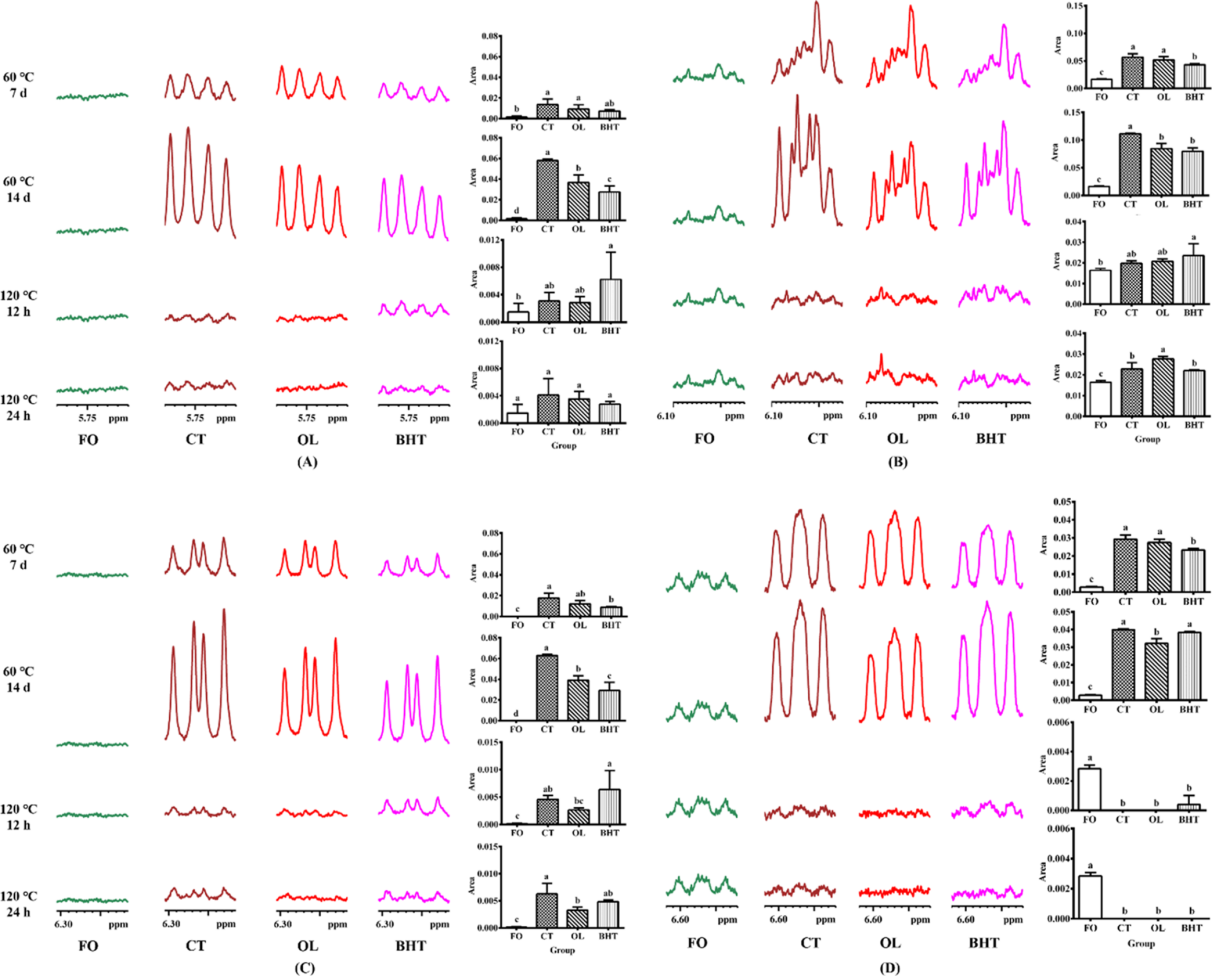
Amplified ^1^H NMR spectra of sunflower oil at different temperatures (60 °C, 120 °C) at different chemical shifts of 5.71–5.78 ppm (A), 5.95–6.1 ppm (B), 6.215–6.305 ppm (C), and 6.52–6.62 ppm (D). **FO**: fresh unheated sunflower oil; **CT**: control sunflower oil; **BHT**: sunflower oil with addition of 200 ppm butylated hydroxytoluene; **OL**: sunflower oil with addition of an equimolar amount of octacosanol lipoate.

Conjugated dienes are another major oxidation product. As shown in Figure 12, the peaks with chemical shifts of 5.71–6.62 ppm were all proton signals of conjugated dienes. Similar to hydroperoxides, the signals of conjugated dienes were almost invisible in FO, but their signal intensity increased with prolonged heating at 60 °C. After 14 days of heating at 60 °C, the proton signal intensity in the OL group was significantly lower than that in the CT group, with decreases of 36.65, 23.94, 37.84, and 19.17%, respectively, while the BHT signal intensity at 6.52 and 6.62 ppm was not significantly different from that in the CT group. After the mixture was heated at 120 °C for 24 h, the proton signal intensity at 6.215–6.305 ppm in the OL group decreased by 47.87% compared to that in the CT group, while BHT had no effect. This indicates that OL had a good inhibitory activity on the formation of conjugated diene when heating sunflower oil at a high temperature.

It is well-known that lipid oxidation commences with the formation of primary oxidation products, encompassing hydroperoxides and conjugated dienes. These primary products subsequently degrade to yield secondary oxidation products, such as low-molecular-weight aldehydes, ketones, and acids. In our previous study, we observed significant signals for aldehydes and epoxides at 9.5–10.2 and 2.9–3.2 ppm, respectively.^23^ Nevertheless, in this study, no signals corresponding to these secondary oxidation products were observed in the ^1^H NMR spectra of the heat-treated sunflower oil. This can be attributed to several factors: first, the oxidation conditions in this study differ from our previous study, which involved open test tubes and more severe oxidation conditions, leading to the production of greater quantities of secondary oxidation products. Second, aldehydes, ketones, and acids are highly volatile, making it challenging for them to remain stable within the oxidized oil. Furthermore, the array of secondary oxidation products is extensive, and the relative content of each individual product is very low, which complicates their detection.

In this study, we successfully synthesized octacosanol lipoate for the first time using octacosanol as the acyl acceptor and *Candida sp*. 99–125 lipase as the catalyst. Under optimal conditions, the conversion rate of octacosanol lipoate could reach as high as 98.1% within 2 h, with an overall yield of 87.9%. The hydrophobicity of lipoic acid was significantly enhanced when esterified with octacosanol. Both traditional methods and ^1^H NMR analysis consistently demonstrated that octacosanol lipoate exhibited superior antioxidant activity compared to BHT at high temperatures. Therefore, octacosanol lipoate is expected to be developed into a safe and efficient natural antioxidant that can be utilized not only in daily cooking oils but also in frying oils.
